# Genome-Wide Characterization and Comprehensive Analysis of NAC Transcription Factor Family in *Nelumbo nucifera*

**DOI:** 10.3389/fgene.2022.901838

**Published:** 2022-06-08

**Authors:** Heyun Song, Yanling Liu, Gangqiang Dong, Minghua Zhang, Yuxin Wang, Jia Xin, Yanyan Su, Heng Sun, Mei Yang

**Affiliations:** ^1^ Key Laboratory of Plant Germplasm Enhancement and Specialty Agriculture, Wuhan Botanical Garden, Chinese Academy of Sciences, Wuhan, China; ^2^ University of Chinese Academy of Sciences, Beijing, China; ^3^ Aquatic Plant Research Center, Wuhan Botanical Garden, Chinese Academy of Sciences, Wuhan, China; ^4^ Amway (China) Botanical R&D Centre, Wuxi, China

**Keywords:** lotus, NAC transcription factor, transactivation, development, stress

## Abstract

NAC (NAM, ATAF, and CUC) is a ubiquitously expressed plant-specific transcription factor (TF) family which is involved in the regulation of various biological processes. However, a systematic characterization of *NAC* gene family is yet to be reported in lotus. Here, 82 *NnNAC* genes which included five predicted membrane-bound NAC proteins were identified in the lotus genome. Phylogenetic analysis revealed seven-subfamily clusters (I–VII) of NnNAC proteins, with homologous gene pairs displaying similar conserved motifs and gene structure characteristics. Transactivation assay of NnNAC proteins revealed an extensive transcriptional activation capacity which is mediated by the highly divergent C-terminal activation domain (AD). Expression analysis of *NnNAC* genes in lotus tissues showed high transcript levels in root, stamen, petal and seed coat. In addition, 30 and 29 differentially expressed *NnNAC* candidate genes putatively involved in lotus seed development and response to complete submergence stress, respectively, were identified. Overall, our study provides potentially useful candidate gene resources for future molecular breeding of lotus varieties with novel agronomic traits.

## Introduction

Transcription factors (TFs) are master regulators of gene expression. NAC is one of the largest and most characterized plant-specific TF superfamily with 117 and 152 members in the dicotyledonous *Arabidopsis thaliana* and *Glycine max*, respectively, and 151 members in the monocotyledonous *Oryza sativa* (Nuruzzaman et al., 2010; Dung et al., 2011; Yan et al., 2017; Li et al., 2018a). NAC protein generally harbors a highly-conserved N-terminal DNA-binding domain (BD) and a highly variable C-terminal transcriptional regulatory region (TRR) (Puranik et al., 2012). The DNA binding of NAC TFs is determined by the presence of NAC domains, which are classified into five subdomains designated, A to E. The highly conserved C and D subdomains are necessary for DNA interaction, subdomain A is necessary for protein dimerization or heterodimerization, whereas the highly variable subdomains B and E determine the functional diversity of NAC proteins (Puranik et al., 2012). The highly divergent C-terminal TRRs are responsible for transcriptional regulation (Christiansen and Gregersen, 2014; Lindemose et al., 2014). Most NAC TFs are located in the cell nucleus; however, some membrane-associated NAC proteins show subcellular localization at the endoplasmic reticulum or plasma membrane, which could be attributed to the α-helical transmembrane (TM) motif in the C-terminus (Seo et al., 2010; Li et al., 2016). Membrane-bound *NAC* genes have previously been reported in other species, such as *A. thaliana* (*Arabidopsis*), *O*. *sativa* (rice), and *S. lycopersicum* (tomato) with 13, 5, and 13 gene members, respectively (Kim et al., 2010a; Bhattacharjee et al., 2017). Membrane-bound *NAC* genes have important regulatory functions in stress responses and are strictly post-translationally regulated under specific conditions (Dung et al., 2011).

The first reported *NAC* gene developed no shoot apical meristem (SAM) in Petunia embryos and showed an equiformal phenotype in SAM formation in *Arabidopsis* mutants (Souer et al., 1996; Takada et al., 2001). The NAC TF family has been associated with various biological processes, including tissue development, hormone response, organogenesis, secondary cell wall (SCW) biosynthesis, and stress response (Zhong et al., 2006; Yamaguchi et al., 2010; Nakano et al., 2015; Yuan et al., 2020). In addition to regulating these processes, numerous NAC TFs have been shown to modulate seed development in some plant species. For example, three seed-size related *NAC* genes, *ONAC020*, *ONAC023*, and *ONAC026* with varied expression levels among rice accessions were highly expressed during seed development (Mathew et al., 2016). Similarly, *ONAC025*, *ONAC127*, and *ONAC129* were found to be involved in seed development by modulating rice grain filling during reproductive period (Mathew et al., 2020; Ren et al., 2021). Plants constantly encounter diverse biotic and abiotic stresses throughout their lives, which cause adverse effects on their growth and development. Increasing evidence suggests that NAC proteins play pivotal roles in abiotic stress tolerance (Puranik et al., 2012; Shao et al., 2015; Yuan et al., 2020; Singh et al., 2021). In *Arabidopsis*, *ANAC019*, *ANAC055*, and *ANAC072* are transcriptional activators of drought stress response, and their overexpression improved drought tolerance in transgenic plants (Tran et al., 2004). Similarly, a *NAC SlJUB1* gene, was shown to enhance tomato drought tolerance by activating the expression of several stress related genes, such as *SlDREB1*, *SlDREB*, and *SlDELLA* (Thirumalaikumar et al., 2018). Due to their roles in regulating important biological processes, comprehensive identification of stress related *NAC* genes is thus crucial for studying stress response mechanisms in plants.

Lotus (*Nelumbo nucifera* Gaertn.) is an old domesticated perennial wetland plant in the family Nelumbonaceae, which contains a single genus, *Nelumbo*, with two extant species: *N. nucifera* Gaertn. and *N. lutea* Pers. (Ming et al., 2013; Sun et al., 2021). The lotus seeds, rhizomes, and flowers have versatile uses, including as popular vegetable and ornamental plant with diverse medicinal properties (Sun et al., 2020). Lotus is popularly cultivated in Asia, and it is mainly categorized as seed-, rhizome-, and flower-lotus based on agronomic traits and distinct uses (Yang et al., 2015). Seed-lotus is predominantly cultivated for its edible lotus seeds, which are rich in proteins, vitamins, minerals, essential amino acids, and other numerous health-promoting bioactive components (Li et al., 2018b; Sun et al., 2021). Rhizome-lotus is produced for its edible underground stems, while flower-lotus is popular for its diversely colored and shaped flowers (Yang et al., 2015). TFs, including MYB, WRKY, bHLH, ERF, and bZIP have previously been reported to play crucial roles in lotus growth and development (Chen et al., 2013; Deng et al., 2016; Li et al., 2018b; Li et al., 2019; Sun et al., 2020). For example, Deng et al. (2016) demonstrated the *NnMYB5*-mediated transcriptional activation of anthocyanin synthesis, and its overexpression induced higher anthocyanin accumulation in immature transgenic *Arabidopsis* seeds and flower stalks. In addition, *NnANT* and *NnAP2* have been reported as potential negative regulators of lotus seed size and development (Li et al., 2018b). Moreover, the lotus *NAC* genes have been implicated in root browning under anaerobic stress and the formation of adventitious roots (Cheng et al., 2019; Min et al., 2019). However, despite previous reports, comprehensive mapping of genome-wide NAC TF family, which could potentially improve our knowledge on the gene family function, distribution, and evolution in sacred lotus is still lacking.

The recently improved sacred lotus genome assembly by Shi et al. (2020) provides an excellent opportunity to investigate the genome-wide distribution and evolution of *NAC* gene family in lotus. This study aimed to conduct a comprehensive analysis to clarify sequence features, phylogenesis, genome synteny, expression patterns, subcellular localization, and transcriptional activation capacity of *NAC* gene family members in sacred lotus. The study also integrated the analysis of possible roles of *NnNAC* genes in lotus seed development and response to complete submergence stress. Our results will not only improve the understanding on *NnNAC* gene family function, genome distribution, and evolution, but also provides vital candidate genes for future molecular breeding in lotus.

## Materials and Methods

### Identification and Phylogenetic Analysis of *NAC* Genes in the Sacred Lotus Genome

To identify *NnNAC* genes, we retrieved the gene annotation gff3 file containing all the predicted protein sequences from lotus reference genome (*N.* Gaertn.) (Li et al., 2021a). The *NnNAC* genes were predicted using PlantTFDB 4.0 (http://planttfdb.cbi.pku.edu.cn/), and their complete amino acid sequences were confirmed with Clustalx v.1.81 and MEME software (http://meme-suite.org/index.html). Predicted molecular weights (Mw) and isoelectric points (pI) of NnNAC proteins were calculated using ExPASy software (http://web.expasy.org/protparam/). Membrane-bound NnNAC members were predicted using the TMHMM server v.2.0 (http://www.cbs.dtu.dk/services/TMHMM/).

Multiple sequence alignments of the identified 82 NnNAC proteins were carried out using Clustalx v.1.81 with default settings. Phylogenetic tree was constructed in MEGA7 using the Neighbor-Joining algorithm, the evolutionary distances were computed with the Poisson correction method, and tree nodes were evaluated with 1,000 bootstrap replications (Kumar et al., 2016).

### Gene Structure, Conserved Motifs, Promoter, Chromosomal Location and Genome Scale Syntenic Analysis of *NnNAC* Genes

To obtain the intron and exon structures, the coding and genomic sequences of *NnNAC* genes were analyzed with the Gene Structure Display Server (GSDS v.2.0) online program (http://gsds.cbi.pku.edu.cn/). Conserved motifs were identified using the MEME online tool (http://meme-suite.org/index.html) with default settings. For promoter sequence analysis, a 2-kb length sequence upstream of the start codon of each gene was downloaded for *cis*-elements prediction using PlantCARE program (http://bioinformatics.psb.ugent.be/webtools/plantcare/html/) (Lescot et al., 2002). Distribution of *NnNAC* genes within the lotus chromosomes was visualized using TBtools software (Chen et al., 2020). *NAC* genes were sequentially named from chromosome (Chr) 1 to 8 with numerical digits in the order of their physical location from top to bottom of the chromosomes.

Genome sequences of *A. thaliana* and *O. sativa* were downloaded from Phytozome v13 (https://phytozome-next.jgi.doe.gov/). Genome scale syntenic analysis between the three plant species was conducted by MCScan X, and TBtools software was used to visualize their relationship (Wang et al., 2012; Chen et al., 2020).

### Expression Profiling of *NnNAC* Genes

To investigate the expression patterns of *NnNAC* genes in lotus tissues, the transcriptome data corresponding to gene expression abundance in various tissues were obtained from Nelumbo Genome Database (http://nelumbo.biocloud.net/nelumbo/home). Public transcriptome datasets on seed development and response to complete submergence stress were retrieved from the National Center for Biotechnology Information (NCBI) with accession numbers, SRP127765 and PRJNA723672, respectively (Li et al., 2018b; Deng et al., 2022). We performed quality control of the downloaded transcriptome data sets with FastQC and Trimmomatics programs (Bolger et al., 2014; Wingett and Andrews, 2018). Subsequently, gene expression levels were quantified by fragments per kilobase of transcript per million mapped fragments (FPKM) using StringTie software (Pertea et al., 2015). Gene expression was visualized using TBtools software. DESeq2 R package v.1.10.1 was used for pairwise comparisons to identify differentially expressed genes (DEGs) with filter the criteria, Fold Change (FC) ≥ 2 and False Discovery Rate (FDR) < 0.05 (Love et al., 2014).

### qRT-PCR Analysis

High-quality RNAs were reverse transcribed to cDNA using TransScript One-Step gDNA Removal and cDNA Synthesis SuperMix (Lot#M31212, Beijing TransGene Biotech, Beijing, China). qRT-PCR analysis was carried out on a StepOnePlus™ Real-time PCR System (Applied Biosystems, United States). The relative gene expression level was calculated and normalized using *NnACTIN* (Gene ID NNU_24864) used as the internal standard. All primers used for qRT-PCR were listed in Supplementary Table S1.

### Subcellular Localization of *NnNAC* Genes

ProtComp v9.0 (http://linux1.softberry.com/berry.phtml) was used to obtain the subcellular localization predictions of NnNAC proteins. Coding regions of *NnNAC* genes were cloned into entry vectors (pDONR221 Zeo) using high fidelity primers (Supplementary Table S1) following the BP-clonase kit instruction manual (Lot#2335893, Invitrogen by Thermo Fisher Scientific, United States). Transformed plasmids were then cloned into PMDC43 vectors with LR-clonase according to the manufacturer’s instructions. These plasmids were subsequently inserted into the *Agrobacterium tumefaciens* cv. GV3101 using electric shock method. Young leaves of tobacco plants (4–6 weeks-old) were selected for transformation assay according to the methods of Kokkirala et al. (2010). A confocal laser scanning microscope (Leica TCS SP2; Leica microsystems, Wetzlar, Germany) was used to photograph the agroinfiltrated leaves 48 h after infiltration.

### Transcriptional Activation Analysis

Transactivation capacity of NnNAC proteins was assessed using yeast transactivation assay (Sun et al., 2018). The full-length coding sequences of *NnNAC* genes and the truncated *NnNAC-AD* and *NnNAC-BD* forms were independently cloned in pGBKT7 vectors. The resulting plasmids were subsequently transformed into Y2H gold strains according to manufacturer’s instructions (Cat.No.630489, Clontech) then plated on SD growth mediums without Trp, but with X-α-Gal (SD-Trp + X-α-Gal) to identify the transactivation activity of NnNAC proteins. SD mediums were photographed after 36 h incubation. Primers used for this assay were listed in Supplementary Table S1.

## Results

### Genome-Wide Identification of *NAC* Genes in the Lotus Genome and Their Gene Structural and Conserved Motifs Analyses

**FIGURE 1:**
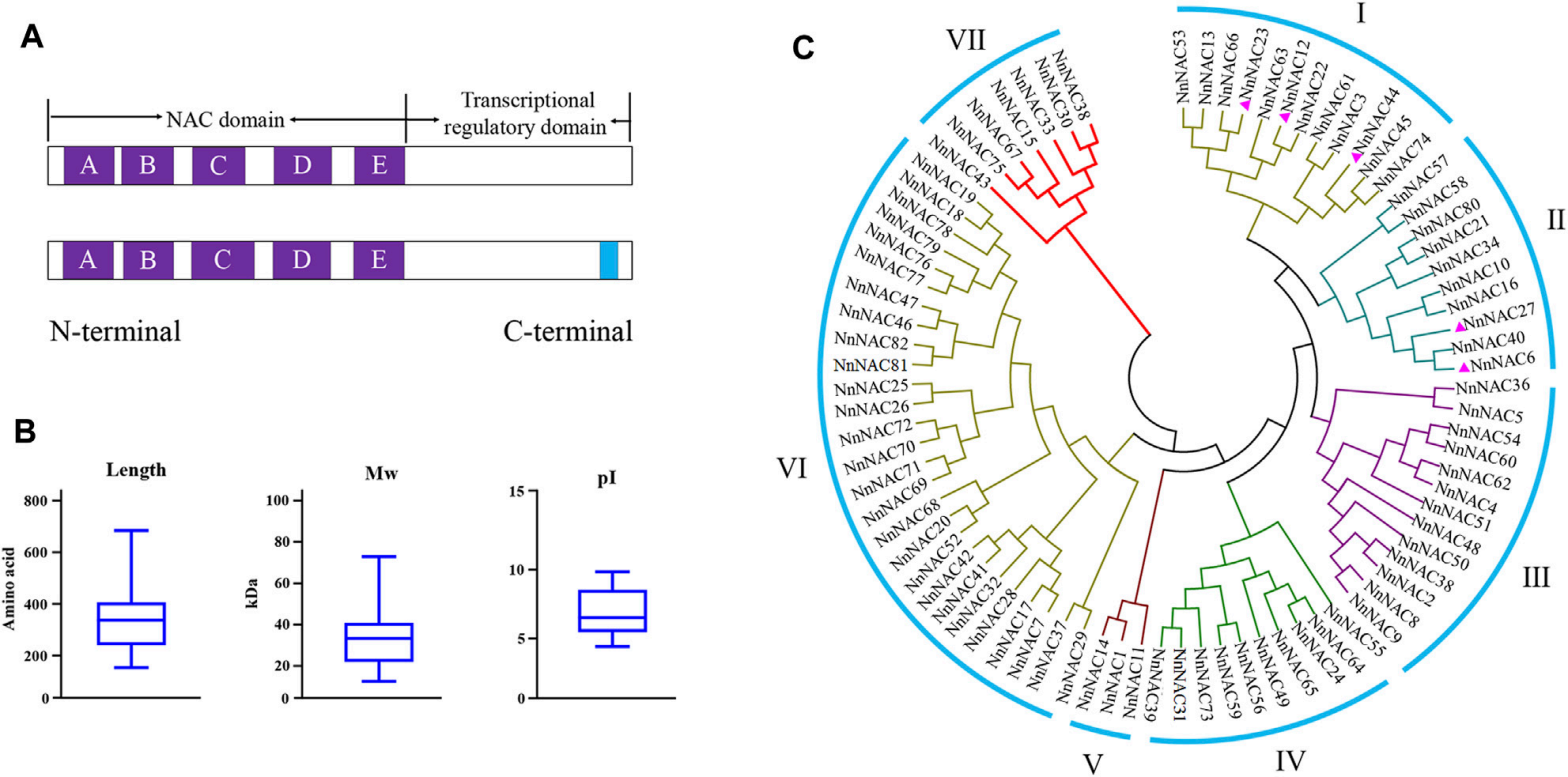
NAC domains, protein sequence features and phylogenetic analysis in lotus. **(A)** Schematic representation of the highly conserved NnNAC key domain and subdomains A–E (purple squares) at the N-terminal. The blue square represents the transmembrane motif (TM). **(B)** Box plots showing statistical distribution of amino acid sequence length, molecular weight (Mw) and isoelectric point (pI) of NnNACs. **(C)** Phylogenetic clustering of 82 identified lotus NAC proteins. The purple triangle represents NnNAC protein harboring the TM motif.

**FIGURE 2:**
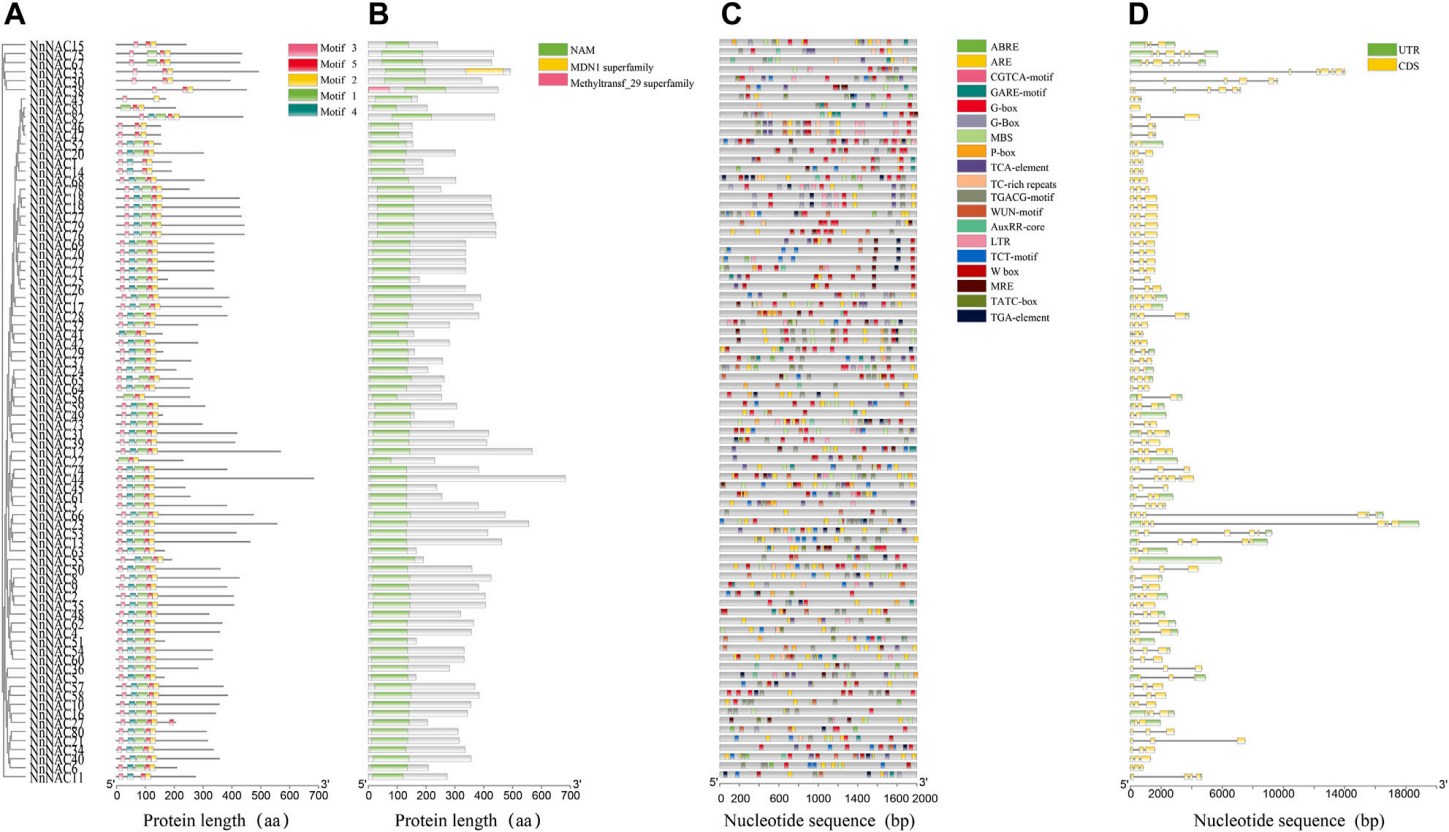
Conserved motifs, *cis*-elements and *NnNAC* gene structure analyses. **(A)** Clusters and conserved motifs of NnNAC proteins. **(B)** Screening of conserved NAC domain in lotus. **(C)** Identification of *cis*-regulatory elements in the 2-kb upstream promoter regions of *NnNAC* genes. **(D)** The lotus *NAC* gene structures.

A total of 82 complete non-redundant *NnNAC* genes were identified in the lotus genome (*N. nucifera* Gaertn.) from PlantTFDB (Supplementary Table S2). Multiple sequence alignment revealed the presence of conserved NAC domain and the five (A–E) subdomains among all predicted NnNAC proteins (Figures 1A, 2B). Five putative membrane-bound NAC proteins each harboring a single predicted TM at the C-terminal were identified, including NnNAC6, NnNAC12, NnNAC23, NnNAC27, and NnNAC44 (Figure 1A; Supplementary Figure S1; Supplementary Table S2). Analysis of NnNAC protein features revealed that their amino acid residue, molecular weight (Mw), and protein isoelectric point (pI) ranged from 152 to 683, 7.63 to 77.94 KDa, and 4.48 to 9.92, respectively (Figure 1B; Supplementary Table S3).

To understand the evolutionary relationships among the *NnNAC* gene family, a phylogenetic tree was constructed using the 82 identified protein sequences. All NnNAC proteins were clustered into seven subfamilies (I–VII) (Figure 1C). Notably, the five predicted membrane-bound proteins were exclusively present in I and II subfamily clusters (Figure 1C). Conserved motif analysis identified five conserved protein motifs, with motif 2 and motif 5 as the most conserved (Figure 2A). Interestingly, NnNACs within the same phylogenetic cluster tended to harbor similar conserved motifs, while paired NnNACs, such as NnNAC1 and NnNAC14, NnNAC30 and NnNAC38, NnNAC46 and NnNAC47, and NnNAC67 and NnNAC75 shared equiformal conserved motifs (Figure 2A).

Gene structure variability due to different exon and intron combinations is useful for understanding the diverse gene functions and genome evolution. To explore the structural diversity of *NnNAC* genes, the exon/intron arrangements were analyzed. The numbers of exons among *NnNACs* genes varied from one to seven, with most genes (64.2%) having three exons (Supplementary Figure S2). *NnNAC38* had seven exons which was the highest number observed, while *NnNAC55* and *NnNAC81* only harbored one exon each (Figure 2D). Notably, genes sharing the same phylogenetic cluster showed similar conserved structural characteristics (Figure 2).

### Chromosomal Location and Syntenic Analysis of *NAC* Genes in Lotus, *Arabidopsis* and Rice

**FIGURE 3:**
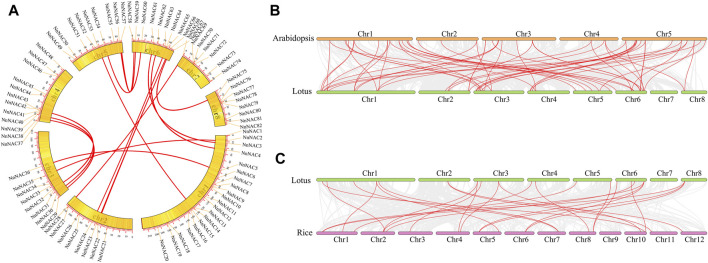
Genome scale synteny analysis of lotus, *Arabidopsis*, and rice *NAC* genes. **(A)** Syntenic relationship of *NnNAC* genes. All 82 *NnNAC* genes are marked according to their chromosome distribution in the lotus genome and syntenic gene pairs are connected with red lines. **(B)** Syntenic pairs of *NAC* genes between *Arabidopsis* and lotus. **(C)** Syntenic pairs of *NAC* genes between lotus and rice. The lotus, *Arabidopsis*, and rice chromosomes are shown in orange, green, and purple bars, respectively. Syntenic gene pairs are linked with red lines.

Chromosomal localization revealed uneven distribution of *NnNAC* genes within the lotus genome with each of the eight lotus chromosomes harboring at least one gene. The highest gene density was observed in Chr 1 with 20 *NnNACs*, representing 24% of total genes identified, while only seven *NnNAC* genes were anchored on Chr 7. In addition, 8, 8, 12, 9, 10, and 8 *NnNAC* genes were anchored on Chr 2, 3, 4, 5, 6, and 8, respectively (Figure 3A; Supplementary Figure S3). Interestingly, some *NnNACs* indicated potential evidence of gene duplication events in the lotus genome (Supplementary Figure S3). For example, *NnNAC18* and its homolog *NnNAC19* located within a 0.77-Mb interval on Chr 1 shared about 99.8% identity (Supplementary Figure S4A). Similarly, four *NnNAC69*, *NnNAC70*, *NnNAC71*, and *NnNAC72* anchored within a 0.41-Mb chromosomal region on Chr 7 shared about 98.8% amino acid sequence identity (Supplementary Figure S4B). In addition, 16 pairs of segmentally duplicated *NnNAC* genes were identified by intragenomic synteny analysis. Highly conserved amino acid sequences were observed among some collinear gene pairs, such as *NnNAC54* and its paralog *NnNAC60* located on Chr 6 which shared about 87.5% amino acid sequence identity, and *NnNAC67* on Chr 6 which shared about 88.5% identity with *NnNAC75* anchored on Chr 8 (Supplementary Figures S4C,D).

Genome synteny analysis is an effective method for inferring genome evolutionary history. The evolution and collinearity of *NAC* gene family was explored using the complete genome sequences of sacred lotus, *Arabidopsis* and rice. As a result, a total of 59 orthologous gene pairs sharing high similarities were identified between *Arabidopsis* to lotus (Figure 3B). In contrast, only 26 orthologous gene pairs were identified between rice to lotus genomes (Figure 3C). These results suggest that lotus, an ancient true dicot, is more likely to be evolutionarily closer to dicots than monocots.

### Screening of *NAC* Gene *Cis*-Elements in Lotus

To study the transcriptional regulation of *NnNAC* genes, *cis*-elements were identified using the PLANTCARE database. As a result, numerous *cis*-regulatory elements linked to stress responses, development, and phytohormone responses were detected in the promoter regions of *NnNAC* genes. Of the 82 gene promoters screened, ARE element, CGTCA-motif, TGACG-motif, G-box, ABRE, and W box were the six most commonly identified *cis*-elements with percentage frequencies of 89, 84.1, 82.9, 76.8, 71.9, and 65.9, respectively (Figure 2C; Supplementary Figure S5). The W box and ABRE are both associated with drought and salt stress responses, while TGACG-motif and CGTCA-motif are involved in phytohormone response and development. These results suggest the likely roles of *NnNAC* genes as crucial stress response and development regulators in lotus.

### Subcellular Localization and Transcriptional Activation Assay of NnNAC Proteins

**FIGURE 4:**
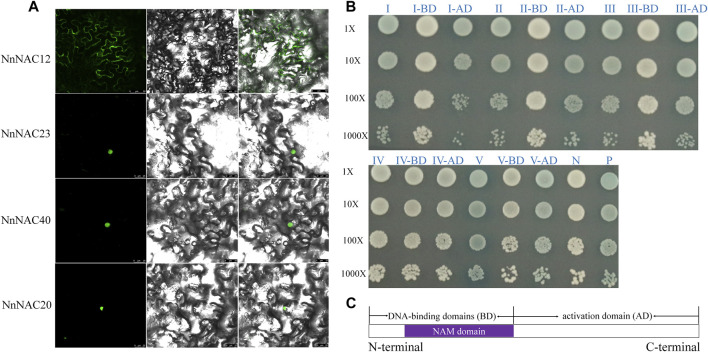
Subcellular localization and transcriptional activation assay of NnNAC proteins. **(A)** Subcellular localization of four NnNAC proteins in tobacco leaves. **(B)** Transactivation assay of NnNAC proteins in the Y2H system. I: NnNAC12; II: NnNAC23; III: NnNAC20; IV: NnNAC52; V: NnNAC40; AD and BD represent NnNAC protein with AD and BD domains, respectively; P: positive control (pGBKT7-53); N: negative control. **(C)** Schematic representation of NnNAC protein truncations.

Bioinformatic studies have predicted that about 74 (∼90%) of the identified NnNAC proteins were nuclear while others were extracellularly located (Supplementary Table S3). Three predicted nuclear located *NnNAC* genes, including *NnNAC20*, *NnNAC23*, and *NnNAC40* and a membrane located *NnNAC12* gene were selected for subcellular localization to test the prediction accuracy results. Microscopic observations showed nuclear localization of NnNAC20, NnNAC23 and NnNAC40, while the GFP signals of NnNAC12 were observed in plasma membranes (Figure 4A).

Transcriptional activation assay is useful for determining the regulatory capacity of TFs. Transactivation capacities of NnNAC proteins were determined using the yeast two-hybrid system (Y2H). Full-length cDNA of five selected NAC proteins, including NnNAC12, NnNAC20, NnNAC23, NnNAC40, and NnNAC52 were individually cloned into GAL4DB vector. As a result, blue colonies indicating transcriptional activation activity were observed in all tested NnNAC proteins except NnNAC52 (Figure 4B). To investigate the transactivation domain, we further truncated the NnNAC proteins into conserved N-terminal DNA-binding domains (BD) and high divergent C-terminal activation domain (AD) regions (Figure 4C). Expectedly, transactivation activity was observed for the AD domain and not for BD domain (Figure 4B).

### Expression Profiling of *NnNAC* Genes in Lotus Tissues

**FIGURE 5:**
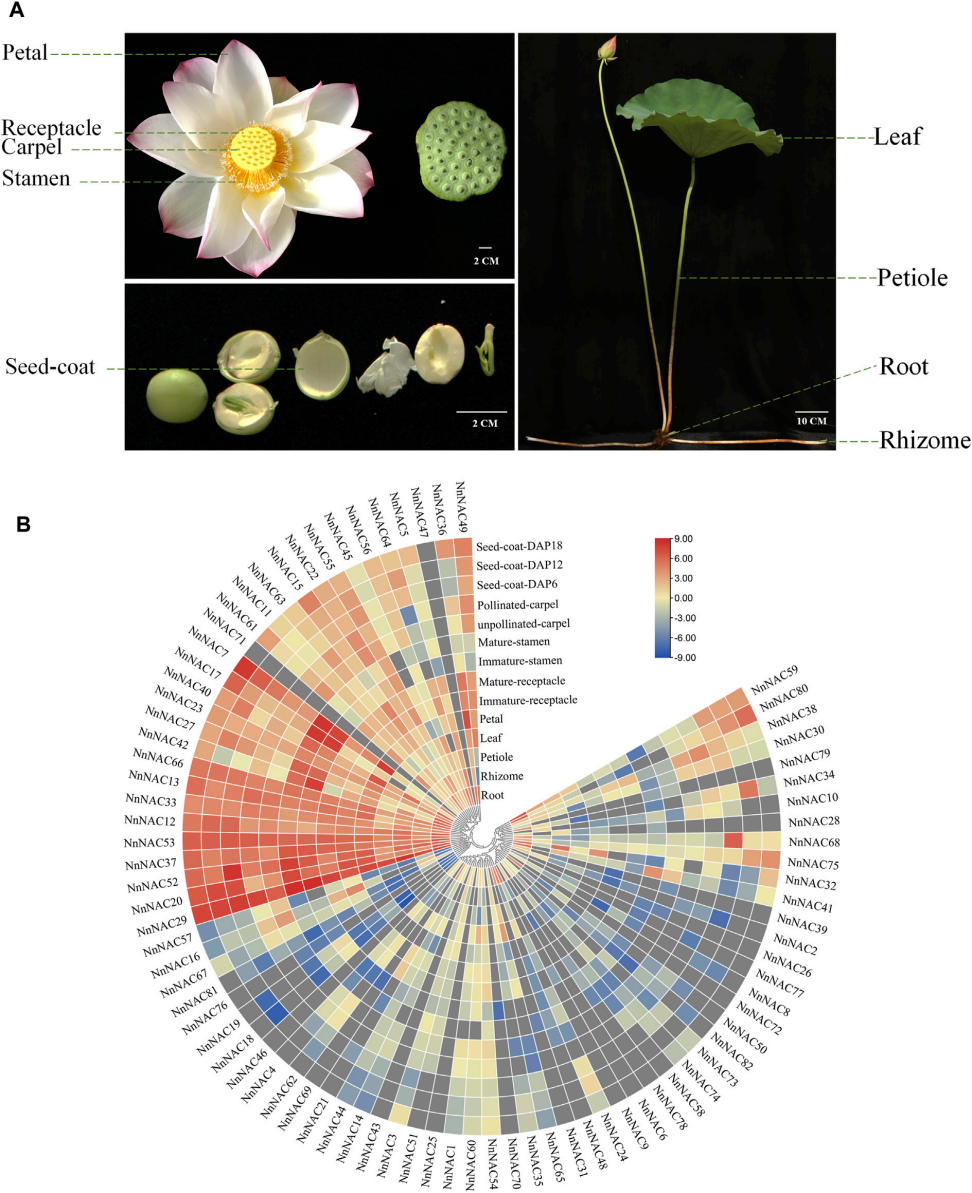
Expression profiling of *NnNAC* genes in different lotus tissues. **(A)** Lotus tissues used to analyze the expression of *NnNAC* genes. **(B)** Heat-map clustering based on FPKM expression of 82 *NnNAC* genes in different lotus tissues.

Published transcriptome data of leaf, petiole, petal, receptacle (immature and mature), stamen (immature and mature), carpel (unpollinated and pollinated), seed coat (6, 12, and 18 days after pollination, DAP), root and rhizome were obtained and used to investigate the tissue expression patterns of *NnNAC* genes (Figure 5A). Overall, 64 (∼78%) of the 82 *NnNAC* genes were expressed in at least one lotus tissue, whereas 14 (∼17%) were expressed in all tested tissues (FPKM ≥ 1). Low transcript abundance of FPKM < 5 in all tested tissues was observed in 42 (∼51%) *NnNAC* genes. Conversely, the expressions of *NnNAC25*, *NnNAC43*, *NnNAC46*, *NnNAC47*, *NnNAC71*, and *NnNAC72* were not detected in all analyzed tissues (Supplementary Table S4). Notably, highly expressed *NnNAC* genes with FPKM ≥ 20 were identified in root, stamen, petal, and seed coat (Figure 5B; Supplementary Table S4). *NnNAC* genes, including *NnNAC24*, *NnNAC31*, *NnNAC41*, *NnNAC48*, and *NnNAC50* exhibited tissue-specific expression in roots, while *NnNAC27* and *NnNAC42* were only expressed in root and stamen. In contrast, *NnNAC20*, *NnNAC29*, *NnNAC37*, *NnNAC52*, *NnNAC53*, and *NnNAC66* were expressed in all tested tissues. Interestingly, phylogenetically close *NnNAC* genes, including *NnNAC24/64*, *NnNAC31/39*, and *NnNAC44/45* exhibited comparable expression patterns. In addition, duplicated gene pairs, such as *NnNAC18/19* and *NnNAC69/70/71/72* also shared similar expression patterns.

### Identification of *NnNAC* Genes Involved in Lotus Seed Development and Complete Submergence Stress Response

**FIGURE 6:**
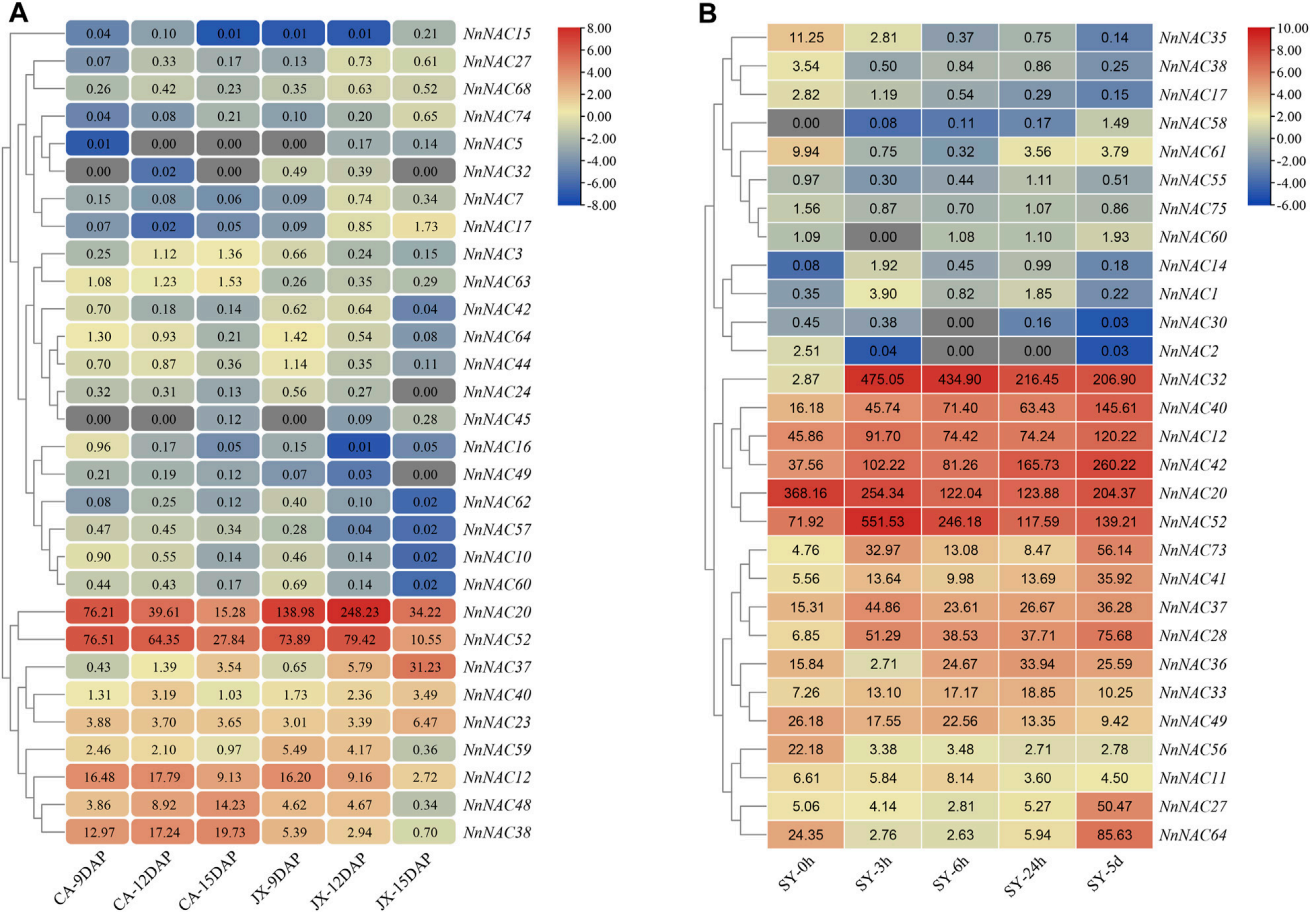
Expression analysis of *NnNAC* genes during lotus seed development and response to complete submergence. **(A)** Heat-map showing the expression patterns of 30 *NnNAC* genes involved in the lotus seed development (FPKM values). **(B)** Heat-map showing the expression patterns of 29 complete submergence stress responsive *NnNAC* genes in lotus (FPKM values). “SY” represent complete submergence stress treatment.

Multiple complex pathways respond to different plant signals in a coordinated manner that involves the recruitment of various transcription factors (Li et al., 2018b; Sun et al., 2020). The published transcriptome data of seed cotyledons at 9, 12 and 15 DAP in “China Antique” (CA) and “Jianxuan 17” (JX) were used to investigate the possible roles of *NnNACs* in lotus seed development. A total of 10 and 23 *NnNAC* DEGs were subsequently identified in CA and JX, respectively, while 20 *NnNAC* DEGs were identified between CA and JX. Overall, 30 *NnNAC* DEGs were identified during lotus seed development (Supplementary Table S5). Of these, 14 (46.7%) *NnNACs* were expressed in the cotyledon in at least one development stage (FPKM ≥ 1) (Figure 6A). Highly expressed *NnNAC* genes, including *NnNAC12*, *NnNAC20*, *NnNAC37*, *NnNAC38*, *NnNAC48*, and *NnNAC52* with FPKM abundance ≥10, and with varied expression patterns during lotus seed development were identified, suggesting their likely roles in the regulation of lotus seed development. The expression of *NnNAC38* showed a continuous increase from 9 to 15 DAP in CA, while it was downregulated in JX. In contrast, the expression profile of *NnNAC37* showed a continuous increase from 9 to 15 DAP in both CA and JX. Notably, preferential expression of *NnNAC20* and *NnNAC52* gene pairs over other *NnNACs* was observed, with the latter showing a continuous decrease in expression from 9 to 15 DAP in CA. In contrast, the expression of *NnNAC20* showed a continuous downregulation in CA, whereas it was upregulated in JX from 9 to 12 DAP. Four *NnNAC* genes, including *NnNAC23*, *NnNAC37*, *NnNAC38*, and *NnNAC40* were randomly selected to evaluate the reliability of RNA-Seq by qRT-PCR. The results showed comparable expression changes with of transcriptome data (Supplementary Figure S6). For example, qRT-PCR and FPKM expression results showed that the levels of *NnNAC37* were persistently elevated from 9 to 15 DAP in both CA and JX. *NAC* genes have previously been implicated in the regulation of various stress signaling pathways. As a perennial aquatic plant, lotus experience frequent submergence stress during its life cycle due to flooding (Deng et al., 2022). To determine potential submergence responsive *NnNACs*, the published transcriptome of completely submerged “Qiu Xing” at different time intervals (0, 3, 6, and 24 h and 5 days) was analyzed. As a result, a total of 29 *NnNAC* DEGs were identified (Figure 6B; Supplementary Table S6). Seventeen highly expressed *NnNAC* genes with FPKM ≥ 10, and exhibiting two different expression patterns were identified. Of these genes, nine (∼52.9%), including *NnNAC12*, *NnNAC28*, *NnNAC32*, *NnNAC37*, *NnNAC40*, *NnNAC41*, *NnNAC42*, *NnNAC52*, and *NnNAC73* were all significantly upregulated with transcript fold increase ranging from 2 to 165.5 after 3 h complete submergence treatment. For example, a 165.5-fold increase in the expression level of *NnNAC32* compared to that of control was observed after 3 h complete submergence, suggesting that it could be a crucial complete submergence stress responsive regulator in lotus. On the contrary, four *NnNAC* genes, *NnNAC20*, *NnNAC35*, *NnNAC49*, and *NnNAC56* showed decreased expression profiles after complete submergence treatment, suggesting their unlikely involvement in the lotus submergence stress response. Taken together, these results provide important possible candidate NAC regulators for studying the mechanisms of seed development and response to complete submergence in lotus.

## Discussion

TFs function as important switches of transcriptional networks that accurately regulate gene expression (Jiang et al., 2016; Droge-Laser et al., 2018; Kumar et al., 2021). NAC TF family has been identified as one of the largest plant-specific regulators involved in diverse biological processes of numerous plant species, such as *Arabidopsis*, rice, tomato, and maize (Christiansen and Gregersen, 2014; Su et al., 2015; Singh et al., 2021). However, despite their crucial roles and the availability of complete genome sequence, a comprehensive characterization of *NAC* gene family has not been conducted in the aquatic lotus plant.

### Identification and Sequence Characteristics of *NAC* Gene Family in Lotus

Previous studies have reported that members of the NAC TF family are widely distributed in lower and higher plants, such as moss with 31 members, and *Arabidopsis*, rice, soybean, wheat, tomato, grape, and cucumber with 117, 151, 269, 263, 101, 70, and 83 members, respectively (Nuruzzaman et al., 2010; Xu et al., 2014; Ma et al., 2018; Singh et al., 2021). Our study identified for the first time 82 *NnNAC* genes in the published genome of sacred lotus, and this number is very close to that of cucumber. As a basal eudicot, lotus experienced a whole genome duplication (WGD) event about 18 million years ago (Ming et al., 2013; Wang et al., 2013). Consequently, genes associated with signal transduction might have been preferentially retained during WGD to form the present NAC TF family (De Smet et al., 2013). Despite wide variations in gene length, predicted protein molecular weight, isoelectric point, and exons/introns organization among the identified *NnNAC* genes, they remain relatively conserved with about 64% of the genes having three exons (Figure 2D; Supplementary Figure S2). Similar observations were also made in *Arabidopsis*, rice, tomato, tobacco, and maize (Nuruzzaman et al., 2010; Peng et al., 2015; Su et al., 2015; Li et al., 2018a; Li et al., 2021b; Singh et al., 2021). Homologous *NnNAC* gene pairs predominantly shared higher degree of similarities in predicted protein features, gene structures, and conserved protein motifs, which is consistent with previous findings that duplicate genes derived from a common ancestor evolve independently at the same rate with few variations (Zhang and Wang, 2005; Wang et al., 2010; Rinerson et al., 2015). Membrane-bound NAC TFs have been identified in various plant species, providing direct evidence of their involvement in many biological processes (De Clercq et al., 2013; Li et al., 2016; Bhattacharjee et al., 2017). Here, we identified five membrane-bound NAC TFs which showed over-representation in subfamilies I and II in the lotus genome (Figure 1C; Supplementary Figure S1). All the five genes belonged to the classical membrane-bound NAC TFs, harboring a single α-helical TM located at the C-terminal, which is consistent with the membrane-bound NACs identified in *Arabidopsis* and rice (Kim et al., 2007; Ng et al., 2013). Interestingly, a divergent form of NAC protein with two TM located in front of the NAC domain has been reported in tomato and cotton, indicating evolution and functional differentiation (Olsen et al., 2005; Bhattacharjee et al., 2017; Sun et al., 2018). Studies have shown that membrane-bound NAC TFs could be activated by specific signal on the cell membrane (Kim et al., 2007; Seo et al., 2008). In this study, a predicted NAC protein with TM, NnNAC23, was localized in the nucleus, suggesting that this gene might be regulated by post-translation and being cut off the transmembrane domain.

### Functional and Evolutionary Relationship Assessment of *NnNACs* Using Gene Expression Patterns

Plenty of studies have reported the ubiquitous distribution of NAC TFs in the plant kingdom and their association with various biological processes (Kim et al., 2010b; Nakano et al., 2015; Yuan et al., 2020). The presence of *cis*-elements is crucial for the expression of *NAC* TF genes and their functions. Here, we comprehensively identified numerous *cis*-elements with various functions in the promoter region of *NnNAC* genes, suggesting their likely involvement in various biological processes, such as stress responses and regulation of lotus development. For example, classical stress-responsive *cis*-elements, including LTREs, MYB and DREB (Tran et al., 2004; Nakashima et al., 2012; Yan et al., 2017; Yuan et al., 2020), were identified in the promoter region of *NnNAC* genes. These results provide a basis for future studies on the transcriptional regulation mechanisms and functions of *NnNAC* genes.

The expression patterns of a gene family can be used to predict their functions and evolutionary relationships (Wang et al., 2013). Gene expression pattern showed multiple expression of *NnNAC* genes in different lotus tissues. For example, tissue-specific expression in roots was observed in five *NnNAC* genes, including *NnNAC24*, *NnNAC31*, *NnNAC41*, *NnNAC48*, and *NnNAC50*, suggesting their potential association with lotus root development. Conversely, several *NnNAC* genes, such as *NnNAC20*, *NnNAC29*, *NnNAC37*, *NnNAC52*, *NnNAC53*, and *NnNAC66* were expressed in all tested tissues, thus they could be essential for various developmental processes in the aquatic lotus (Figure 5B). Overall, members of the *NnNAC* gene family displayed evidence of functional redundancy and evolutionary diversity in the lotus genome. Although varied expression patterns were observed among *NnNACs*, most homologous gene pairs shared similar expression patterns, for example, *NnNAC31* and *NnNAC39* were uniquely expressed in the root, *NnNAC29* and *NnNAC37* were constitutively expressed in all tested tissues, while *NnNAC18*/*19* and *NnNAC25*/*26* were not expressed in any of the tissues tested (Figure 5B). Interestingly, similar observations were made for *Arabidopsis* and cotton *NAC* genes (Jensen et al., 2010; Sun et al., 2018). Taken together, our results indicated that *NAC* gene family might have undergone expansion through gene duplication during evolution, resulting in functional redundancy of gene members.

### Identification of Putative Candidate *NnNAC* Genes Involved in Lotus Seed Development and Complete Submergence Stress Response

Lotus seed has important commercial, nutritional and medicinal values, and improving its yield and quality is one of the breeding targets today (Sun et al., 2021). Despite previous studies focused on lotus seed development, little is still known about whether *NnNAC* genes are associated with this process (Wang et al., 2016; Li et al., 2018b). In rice, *ONAC020*, *ONAC023*, and *ONAC026* showed high expression level during seed development, and their sequence variations were correlated with seed size (Mathew et al., 2016). Herein, 30 *NnNAC* DEGs during lotus seed development were identified for the first time, with six genes, including *NnNAC12*, *NnNAC20*, *NnNAC37*, *NnNAC38*, *NnNAC48*, and *NnNAC52* being preferentially expressed, suggesting their potential involvement in the regulation of lotus seed development (Figure 6A).

Efforts have been made to clarify the role of NAC TFs in responses to diverse stress, such as drought, salinity and flooding (Puranik et al., 2012; Shao et al., 2015; Yuan et al., 2020; Singh et al., 2021). Lotus is an aquatic plant that experiences frequent submergence stress due to sudden frequent flooding events. Twenty-nine *NnNAC* DEGs associated with submergence stress response were identified in the lotus genome, of which 17 genes showed high expression levels. For example, *NnNAC32*, an orthologous of *Arabidopsis* stress response *AtNAP* (At1g69490) gene (Sakuraba et al., 2015; Seok et al., 2017), was significantly upregulated under complete submergence treatment, which suggest its possible regulatory role in lotus submergence stress response (Supplementary Figure S7). This result might further confirm the functional differentiation of *NAC* gene family. In addition, two lotus homologs, *NnNAC20* and *NnNAC52*, sharing *∼*46% amino acid sequence identity, and orthologous to the *Arabidopsis* stress related *ANAC2* (At1g01720) NAC TF gene, were identified (Lahiri et al., 2021). However, *NnNAC20* and *NnNAC52* showed different expression patterns under complete submergence, suggesting they could be diverging functionally. Notably, thirteen common *NnNAC* genes were differentially expressed during seed development and stress response, indicating they might play multiple biological functions in lotus. This systematic characterization of lotus *NAC* genes in will pave way for future functional analysis to clarify the roles of these candidate genes in lotus.

## Conclusion

Our study identified 82 *NnNAC* genes for the first time in the sacred lotus genome, and conducted a comprehensive sequence characterization, gene structure, expression profiling and transcriptional activation. In addition, *NnNAC* genes involved in lotus seed development and response to complete submergence stress were highlighted. In future, functional characterization of candidate *NnNAC* genes identified in this study is warranted. Our results provide potentially valuable *NnNAC* gene resources for further lotus genetic improvement.

## Data Availability

The datasets presented in this study can be found in online repositories. The names of the repository/repositories and accession number(s) can be found in the article/Supplementary Material.

## Supplementary Material

Supplementary Figure S1 Structures of membrane-bound NnNAC proteins in lotus.

Supplementary Figure S2 Statistical summary of exon numbers in *NnNAC* genes.

Supplementary Figure S3 Chromosome localization of *NnNAC* genes in lotus.

Supplementary Figure S4 Comparison of NAC amino acid sequence similarities.

Supplementary Figure S5
*Cis*-element analysis in the promoters of *NAC* genes in lotus.

Supplementary Figure S6 Validation of gene expression through qRT-PCR.

Supplementary Figure S7 Phylogenetic relationship between NnNAC proteins and AtNAC proteins in *Arabidopsis*.

Supplementary Table S1 Primer sequences used for vector construction and qRT-PCR validation.

Supplementary Table S2 Protein sequences of *NnNAC* genes.

Supplementary Table S3 Statistical analyses of sequence length, molecular weight, isoelectric point, and predicted subcellar localizations for NAC proteins in lotus.

Supplementary Table S4 The expression of *NnNAC* genes in different organs and different tissues in Lotus (FPKM values).

Supplementary Table S5 The expression of *NnNAC* DEGs involved in lotus seed development (FPKM values).

Supplementary Table S6 The expression of *NnNAC* DEGs involved in completely submerged stress response (FPKM values).
