# Supplementary figures and images for: Genome-Wide Characterization and Comprehensive Analysis of NAC Transcription Factor Family in Nelumbo nucifera
Source: Front Genet. 2022 Jun 8;13:901838. doi: 10.3389/fgene.2022.901838 (PMC9214227; doi:10.3389/fgene.2022.901838)

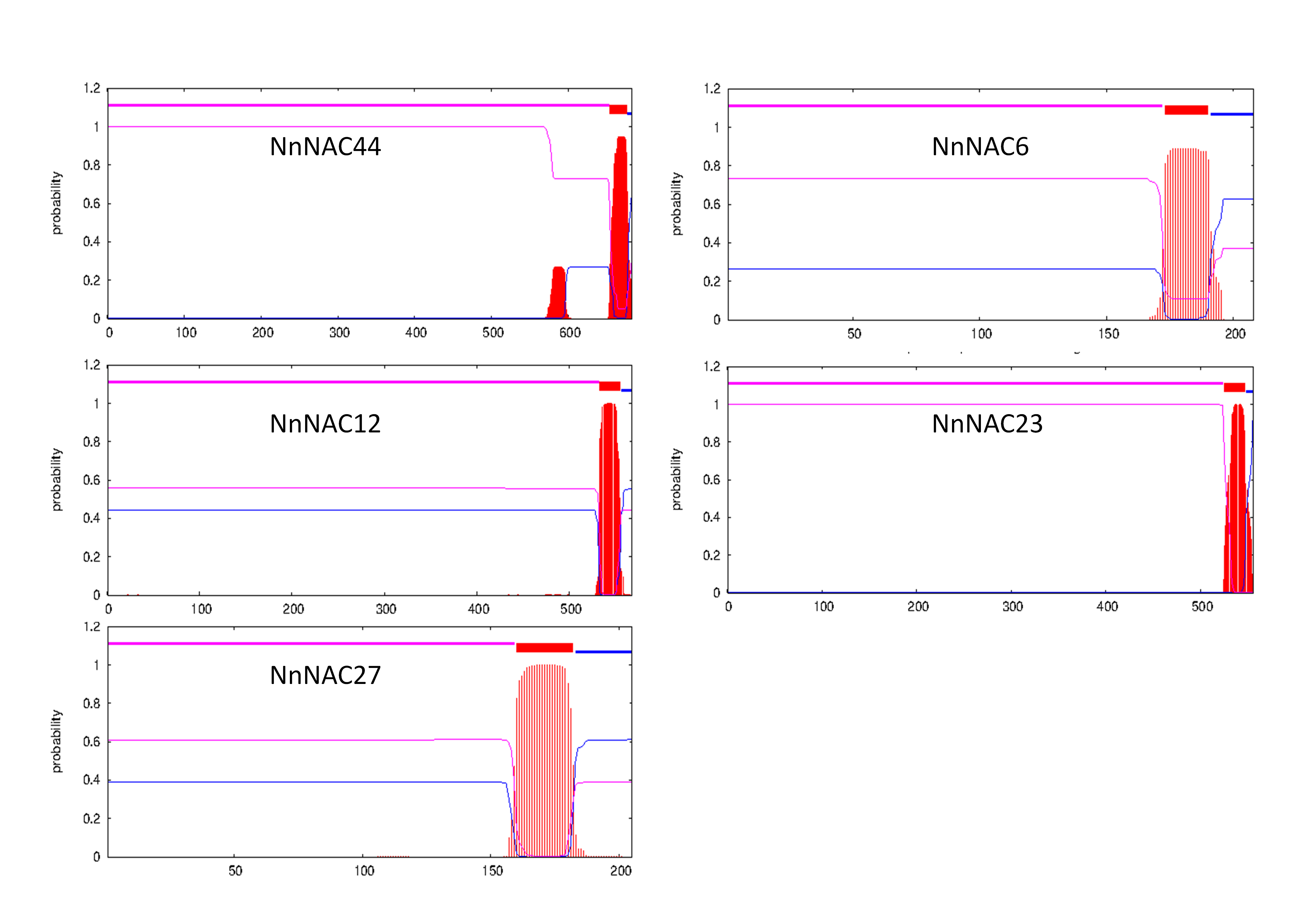

Supplement: Supplementary file 1 [file DataSheet1.ZIP › Supplemental Materials/Fig S1 Structures of membrane-bound NnNAC proteins in lotus.tif]

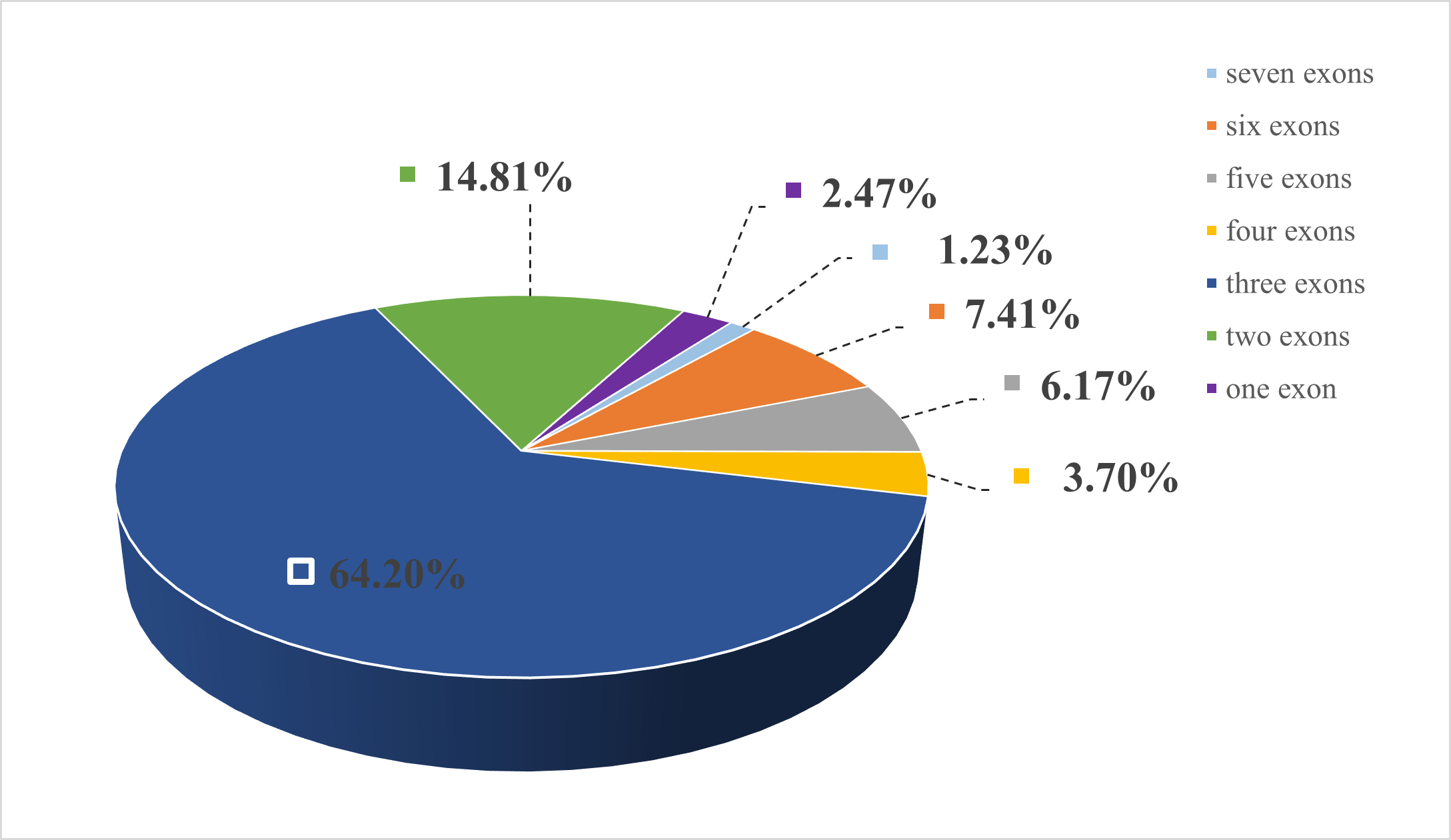

Supplement: Supplementary file 1 [file DataSheet1.ZIP › Supplemental Materials/Fig S2 Statistical summary of exon numbers in NnNAC genes.tif]

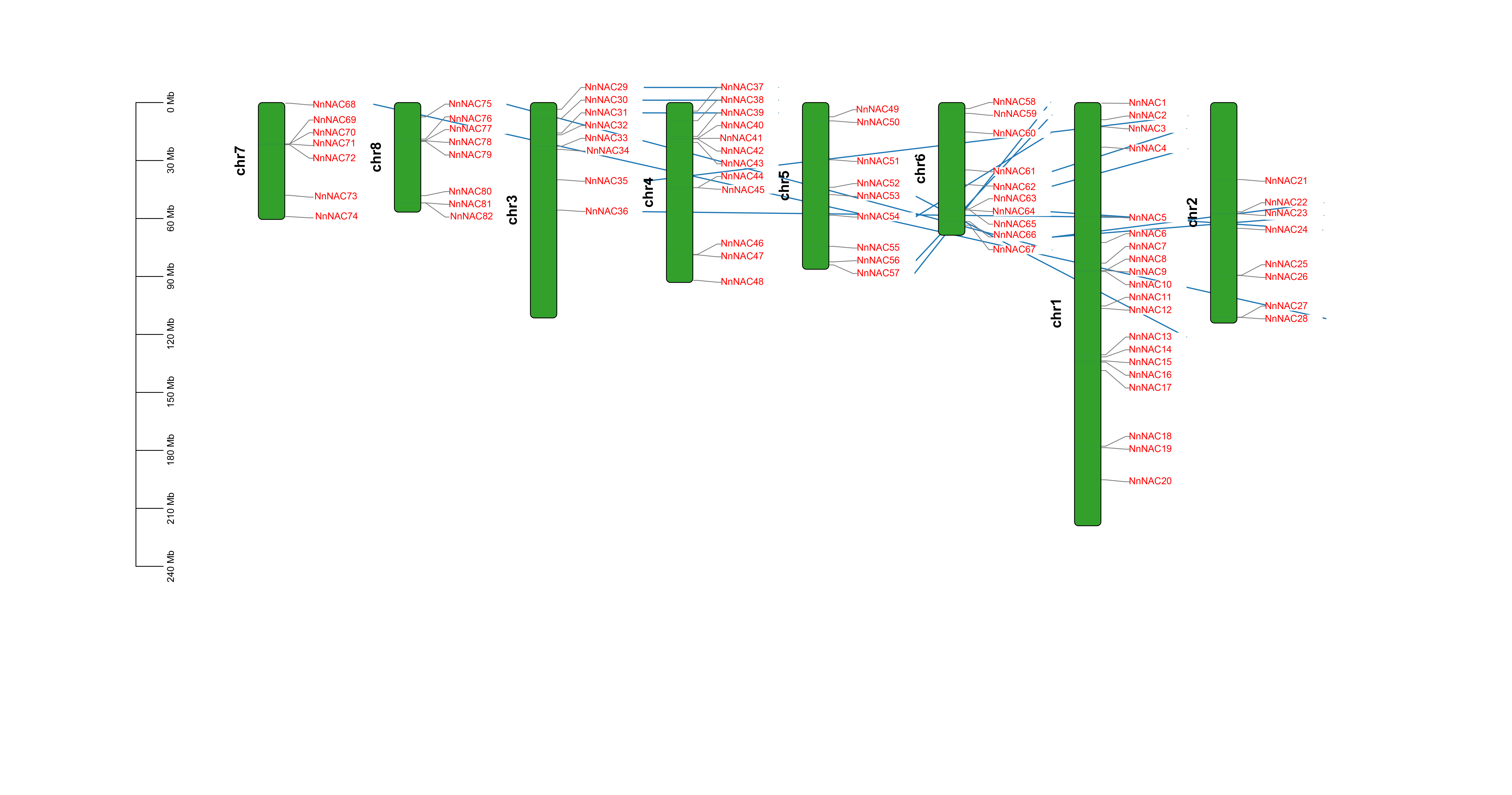

Supplement: Supplementary file 1 [file DataSheet1.ZIP › Supplemental Materials/Fig S3 Chromosome localization of NnNAC genes in lotus.tif]

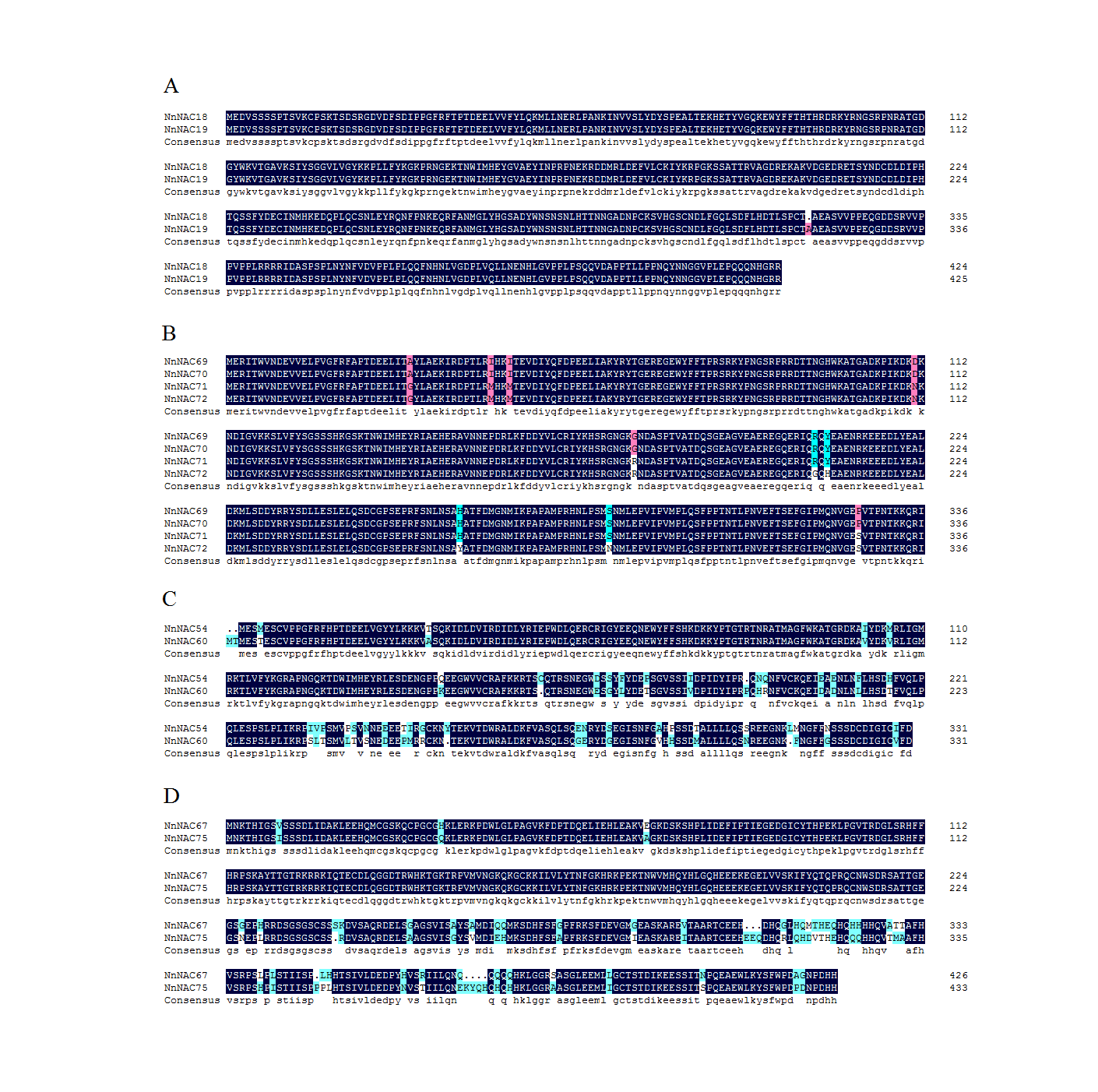

Supplement: Supplementary file 1 [file DataSheet1.ZIP › Supplemental Materials/Fig S4 Comparison of NAC amino acid sequence similarities.tif]

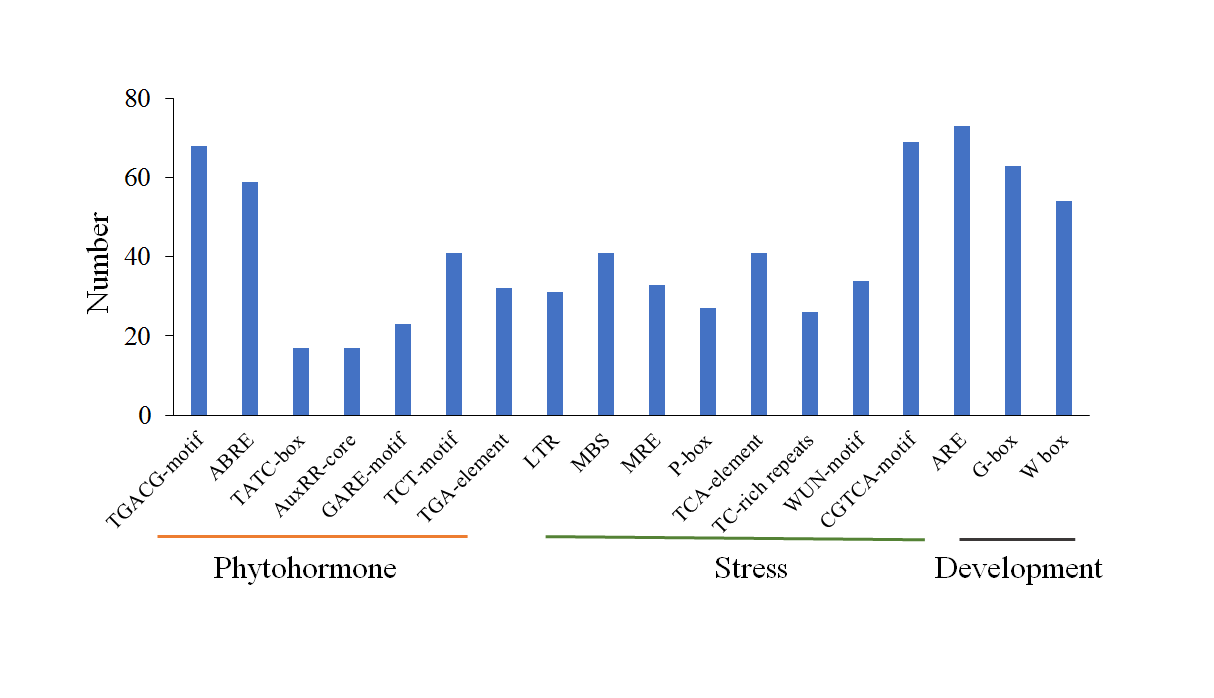

Supplement: Supplementary file 1 [file DataSheet1.ZIP › Supplemental Materials/Fig S5 Cis-element analysis in the promoters of NAC genes in lo.tif]

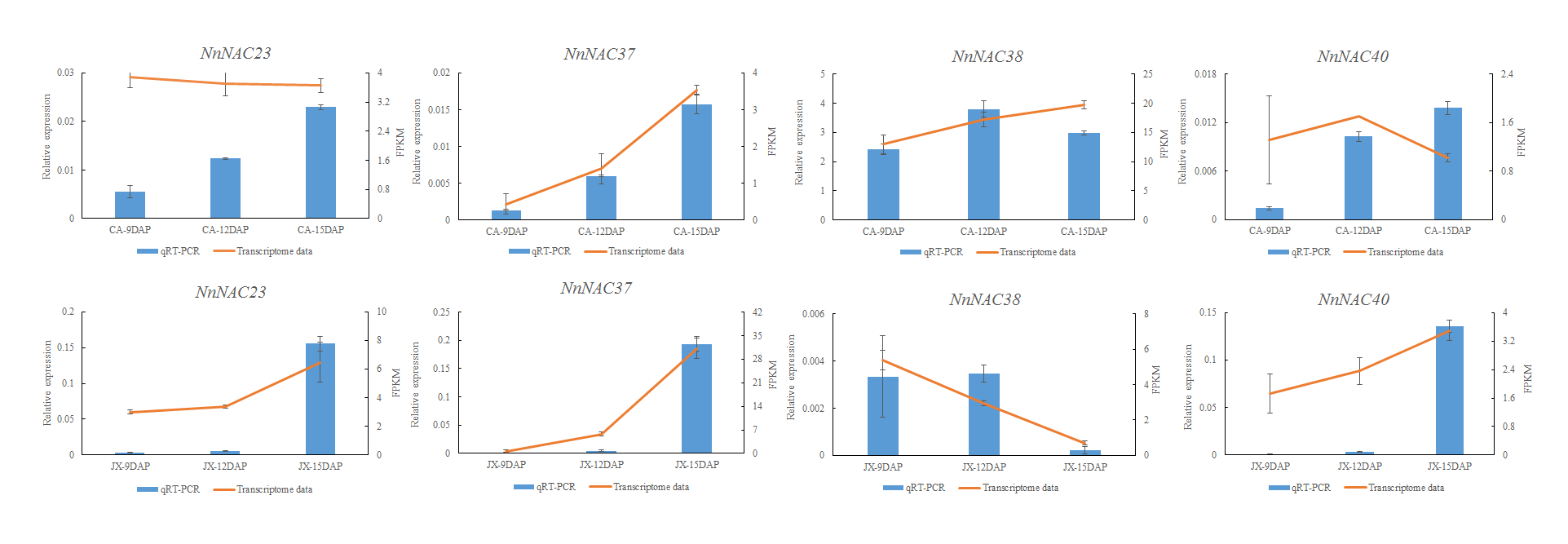

Supplement: Supplementary file 1 [file DataSheet1.ZIP › Supplemental Materials/Fig S6 Validation of gene expression through qRT-PCR.tif]

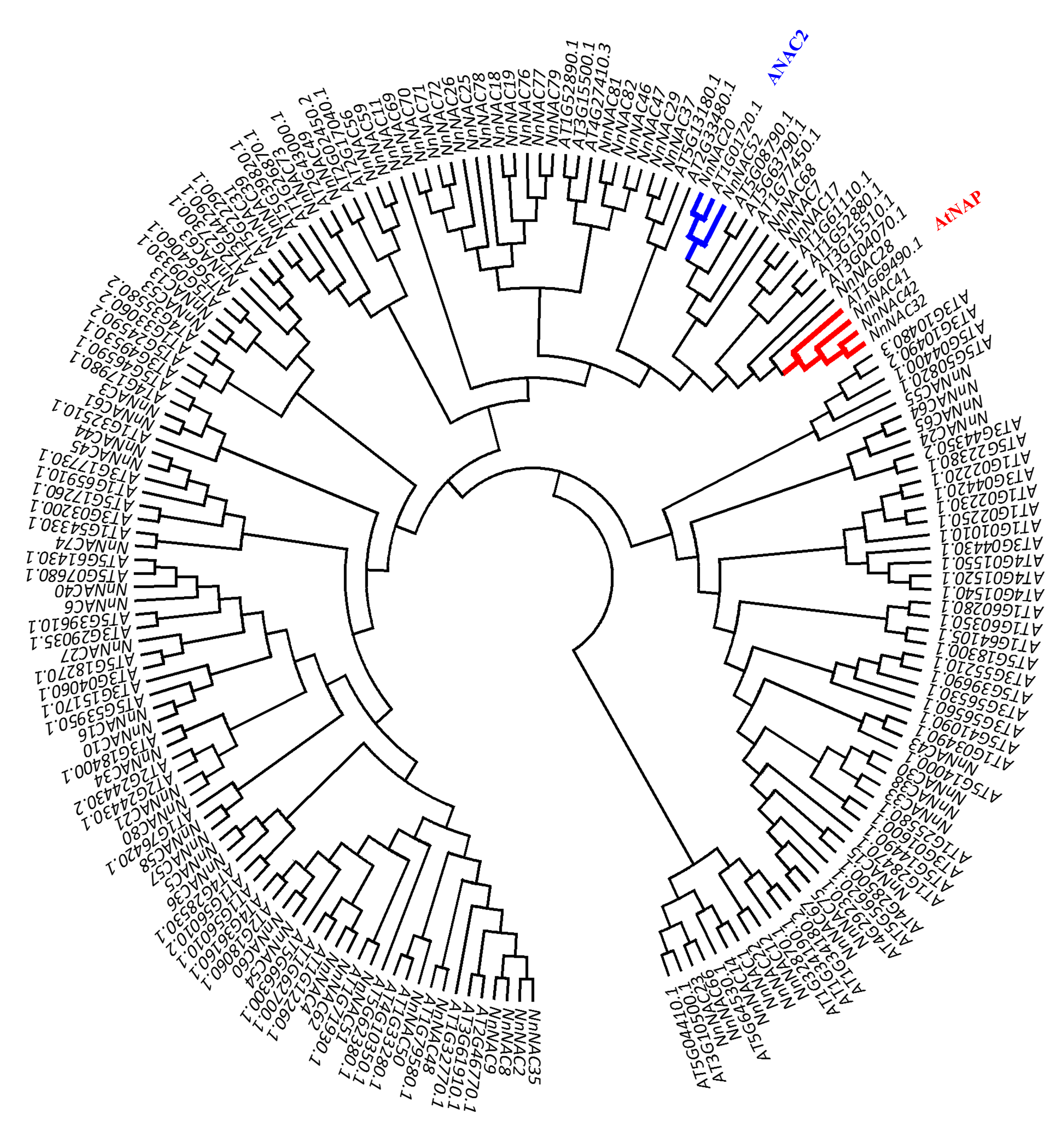

Supplement: Supplementary file 1 [file DataSheet1.ZIP › Supplemental Materials/Fig S7 Phylogenetic relationship between NnNAC proteins and At NAC prot.tif]
